# Assessment of *MBL2* gene polymorphism and lipid peroxidation in Chronic Obstructive Pulmonary Disease (COPD)

**DOI:** 10.1186/1755-8166-7-S1-P95

**Published:** 2014-01-21

**Authors:** Aarti Sharma, Gursatej Gandhi, Jatinder Singh, Sukhdev Singh, Manpreet Kaur

**Affiliations:** 1 Department of Human Genetics, Guru Nanak Dev University, Amritsar, India; 2 Department of Molecular Biology & Biochemistry, Guru Nanak Dev University, Amritsar, India

## Background

Chronic Obstructive Pulmonary Disease (COPD) is fourth leading cause of death worldover. It has been defined as a state characterized by airflow obstruction due to inflammatory reaction. Various innate and adaptive immune system molecules are involved in pathogenesis of COPD. Mannose-binding lectin (MBL) is a Ca^2+^ dependent collagenous lectin which intervene immune response by inhibiting pathogen activity. Point mutations, in codon 54 and 57 of exon 1 of the MBL2 have been reported to affect the serum levels of MBL. Aim of the present study was to investigate the association of MBL2 gene polymorphism with severity and susceptibility towards COPD.

## Methodology

129 COPD patients and 90 age- and sex-matched controls were recruited for the study. Genomic DNA was isolated from blood samples. PCR-RFLP of codon 54 and 57 of the MBL2 were studied using enzymes BanI and MboII respectively. In addition to this, serum MDA concentrations were evaluated by TBA-TCA-HCl method. Genotypic distribution was compared by odds ratio statistics using medcal software. Differences in MDA concentrations were analyzed by student’s‘t’ test using SPSS version 18.0 (IL, USA, and Chicago).

## Results

The genotypic frequencies of codon 54 in COPD patients were significantly higher (p< 0.05) than that of controls. GG genotype was found to be more prevalent in cases (OR= 3.402; CI= 1.14-10.10; p< 0.05). However, there was no significant difference in genotypic distribution for codon 57 of MBL2 gene. Serum MDA concentrations were significantly (p< 0.001) higher in patients (9.00±2.906) as compare to controls (6.31±2.361).

## Conclusion

The results of the present study revealed that MBL2 polymorphism may be involved in pathogenesis of COPD.

